# Novel Mobile Integrons and Strain-Specific Integrase Genes within *Shewanella* spp. Unveil Multiple Lateral Genetic Transfer Events within The Genus

**DOI:** 10.3390/microorganisms10061102

**Published:** 2022-05-26

**Authors:** Teolincacihuatl Ayala Nuñez, Gabriela N. Cerbino, María Florencia Rapisardi, Cecilia Quiroga, Daniela Centrón

**Affiliations:** 1 Instituto de Investigaciones en Microbiología y Parasitología Médica (IMPaM, UBA-CONICET), Facultad de Medicina, Universidad de Buenos Aires, Ciudad Autónoma de Buenos Aires CP1121, Argentina; olincayalan88@gmail.com (T.A.N.); gcerbino@fmed.uba.ar (G.N.C.); mariaflorenciarapisardi@gmail.com (M.F.R.); 2 Laboratorio de Investigación en Biología del ARN Bacteriano IMPaM (UBA/CONICET), Faculty of Medicine, Universidad de Buenos Aires, Ciudad Autónoma de Buenos Aires CP1121, Argentina; 3 Laboratorio de Investigaciones en Mecanismos de Resistencia a Antibióticos IMPaM (UBA/CONICET), Faculty of Medicine, Universidad de Buenos Aires, Ciudad Autónoma de Buenos Aires CP1121, Argentina

**Keywords:** *Shewanella*, chromosomal integron, mobile integron, integrase, gene cassette, lateral genetic transfer

## Abstract

*Shewanella* spp. are Gram-negative bacteria that thrive in aquatic niches and also can cause infectious diseases as opportunistic pathogens. Chromosomal (CI) and mobile integrons (MI) were previously described in some *Shewanella* isolates. Here, we evaluated the occurrence of integrase genes, the integron systems and their genetic surroundings in the genus. We identified 22 integrase gene types, 17 of which were newly described, showing traits of multiple events of lateral genetic transfer (LGT). Phylogenetic analysis showed that most of them were strain-specific, except for *Shewanella algae*, where Son*IntIA*-like may have co-evolved within the host as typical CIs. It is noteworthy that co-existence of up to five different integrase genes within a strain, as well as their wide dissemination to *Alteromonadales, Vibrionales, Chromatiales, Oceanospirillales* and *Enterobacterales* was observed. In addition, identification of two novel MIs suggests that continuous LGT events may have occurred resembling the behavior of class 1 integrons. The constant emergence of determinants associated to antimicrobial resistance worldwide, concomitantly with novel MIs in strains capable to harbor several types of integrons, may be an alarming threat for the recruitment of novel antimicrobial resistance gene cassettes in the genus *Shewanella*, with its consequent contribution towards multidrug resistance in clinical isolates.

## 1. Introduction

*Shewanella* spp. are aquatic bacteria commonly found in a wide variety of marine environments including surface freshwater or the profoundest oceanic trenches [1,2]. A few species, such as *S. algae* and *S. xiamenensis*, can occasionally cause skin and soft tissue, peripancreatic, gastrointestinal and biliary tract infections, otitis, bacteremia, endocarditis, arthritis, peritonitis and ventilator-associated pneumonia [3,4,5]. Genome analyses have revealed that some *Shewanella* spp. may be the reservoirs of antimicrobial resistance determinants, such as *qnr* and *bla*_OXA-48-like_ genes, which were later on transferred to different pathogenic bacteria [3,6,7,8]. In addition, *Shewanella* has a versatile and plastic genome capable of acquiring beneficial genetic traits, such as various antimicrobial resistance gene cassettes encoded within integrons [3,7,9,10].

**Figure 1:**
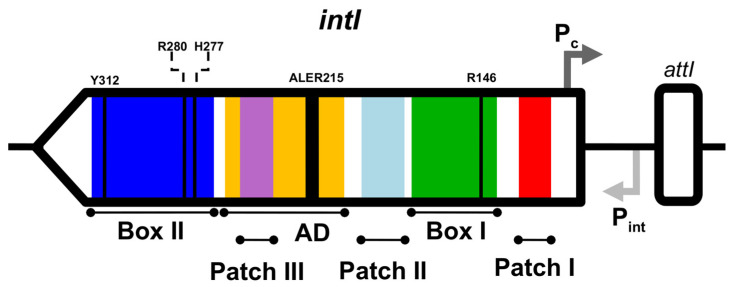
Integron structure. *intI* integrase gene is depicted with a horizontal black arrow; Pc, gene cassette promoter in dark gray, Pint, integrase promoter in light gray; *attI* recombination site with a white rectangle. Colored sections of the integron depict key regions: Box I in green, Box II in blue, Patch I in red, Patch II light blue, Patch III in purple, and the Additional Domain (AD) in yellow. The tetrad RHRY is represented with thin black lines and the conserved motif ALER215 with a thick black line.

Integrons are genetic platforms that participate in the adaptation and evolution of bacteria [11,12,13,14,15] by acquiring and expressing gene cassettes with a wide variety of functions. Several studies showed that integrase genes can be found in around 17% of bacterial genomes deposited in GenBank [12,13,14,15], with as little as 35% amino acid sequence identity, suggesting a long evolutionary history [16]. Integron structure consists of the integrase gene *intI*, a regulatory region (Pint and Pc in class 1 integrons) and an attachment site known as *attI* (Figure 1) [13,16].

Integron integrases are tyrosine recombinases responsible for the integration and excision of gene cassettes, preferably at the *attI* site [13,14,16]. Gene cassettes are mobile elements usually composed of a single structural gene adjacent to an *attC* site, which are recognized and cleaved by the integron integrase by a site-specific recombination mechanism resulting in their integration or excision at the variable region (VR). Once integrated within the VR of the integron, expression of gene cassettes occurs [13,14,16].

Integron integrases possess key residues that define their activity, i.e., R146, H277, R280 and Y312 [17,18]. In addition, they have several conserved residues (Boxes I and II and Patches I, II and III) and a singular additional domain (AD) of about 36 amino acids (near Patch III), with the conserved motif ALER215 and the residue K219 [17,18,19]. Some residues are involved in the catalytic reaction (K171, H277, and G302), while others (E121, W229, and F233) are non-catalytic residues (coordinates assigned based on the IntI1 sequence) [19].

Although there are different schools of thought concerning the classification of integrons [20,21,22,23,24,25,26], they can be classified into two main groups, mobile (MI) and chromosomal integrons (CI). MIs are embedded in mobile genetic elements (MGE), such as transposons, genomic islands or plasmids, that facilitate their dissemination by lateral genetic transfer (LGT) [20,22,23,26]. This group comprises integrons from classes 1 to 5, which are defined by their respective integrase gene [20]. MI integrase genes share between 40–58% sequence identity and some of them can be found in clinical isolates, i.e., *intI1* and *intI2* [3,26]. MIs can harbor up to nine gene cassettes, most of which encode antimicrobial resistance mechanisms to almost all antibiotic families, except for tetracycline and colistin [20,27]. Class 1 integrons have thrived in nosocomial settings, where they can capture and collect antimicrobial resistance (AMR) gene cassettes. This ability is directly linked to the emergence of multi-, extensively or pan-drug resistant bacteria [20,28,29]. On the other hand, CIs are located in the bacterial chromosome and they are proposed to co-evolve with their host over long evolutionary periods of time [20,26,30,31,32,33]. CIs may contain a few and up to 150 gene cassettes depending on the bacterial host and can be found in different environmental species, such as *Nitrosomonas europaea*, most *Vibrio* spp., some *Treponema* spp., *Geobacter sulfurreducens*, and several isolates from the genera *Shewanella*, *Xanthomonas* and *Pseudomonas* [32,34,35,36,37,38]. Most integrases from CIs contain all key residues and motives; however, integrases from *Xanthomonas* spp. have lost their activity due to frameshifts in their gene sequences, interruptions by insertion sequences or deletions [37].

Regarding integron integrases in *Shewanella* genus, previous studies described their presence in *Shewanella oneidensis* MR-1 and in *Shewanella amazonensis* SB2BT, identified as Son*IntIA* and Sam*IntIA*, respectively [34,38]. Sam*IntIA* shared 64.8% sequence identity with Son*IntIA* and 44.6% with IntI1. Furthermore, Sam*IntIA* amino acid sequence analysis showed that it had different residues in the ALER motif [38]. Although both integrases were able to excise antimicrobial resistance gene cassettes at low level frequencies [38], their implication in the threat of antimicrobial resistance requires further studies.

Here, we analyzed the occurrence of integron integrase genes and their genetic surroundings in the genus *Shewanella*, in order to evaluate their association with specific lineages relevant to clinical infections and their contribution with the widespread of antimicrobial resistance gene cassettes. Furthermore, the analysis of our results led us to delve into the evolution and dissemination of chromosomal and mobile integrons.

## 2. Materials and Methods

### 2.1. Shewanella Genomes Dataset and ST Assignment

Our analysis included 304 complete and draft genomes of *Shewanella* spp. retrieved from the Genbank database (https://www.ncbi.nlm.nih.gov/genome, accessed on 1 April 2021) (Table S1). MLST analysis was done using the scheme available at PubMLST website for *Shewanella* spp. (https://pubmlst.org/, accessed on 26 March 2022). Only sequence type (ST) values with 7 or more matches were included in the analysis.

### 2.2. Identification of Integron Integrase Genes and attc Sites

Integron integrase genes were searched in the 304 complete and draft genomes of *Shewanella* spp. with TBLASTN comparative analysis using IntI1 protein (ADW78905.1) as query. Only sequences containing the additional domain were included and any partial IntI protein sequences were excluded from the analysis [25]. Identification of empty integrons (In0), complete integron (integron integrase genes with their respective variable regions), *attC* sites and CALIN elements was done using the software IntegronFinder v2.0 with default setting and the --eagle-eyes option, as recommended by the developers [12]. When necessary, the *attC* sites were analyzed and confirmed using the UNAFold web server [39]. Classification of integron integrases was based on previous nomenclature [13,20,37,38]. Amino acid coordinates were assigned based on the IntI1 sequence from plasmid pVS1 [20].

### 2.3. Pairwise Similarity and Identity Analyses of Integron Integrases

**Figure 2:**
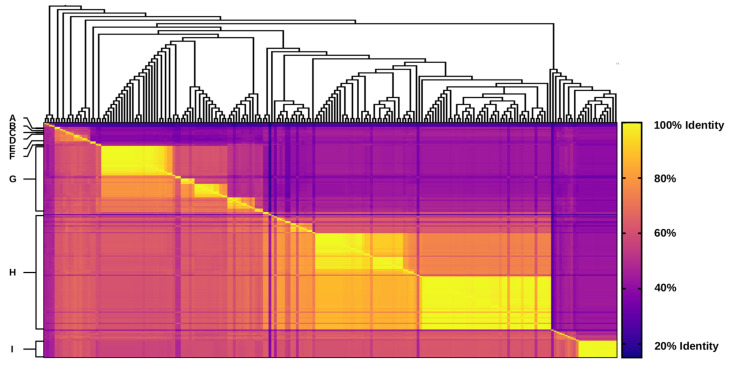
Heat map with identities and similarities of integron integrases from *Shewanella* spp. based on their amino acid sequences. Letters in y-axis and cladogram correspond to the phylogenetic tree and integrase classification from Figure 3. The colored scale depicts the percentages of similarity and identity.

Similarity and identity values of integron integrase protein sequences were calculated using the software MatGAT v2.03 (Matrix Global Alignment Tool) as recommended by the developers [40] (Table S2). Values were plotted as a heat map using GraphPad Prism v8.2.0 software (www.graphpad.com) (Figure 2).

### 2.4. Phylogeny and Sequence Analyses

**Figure 3:**
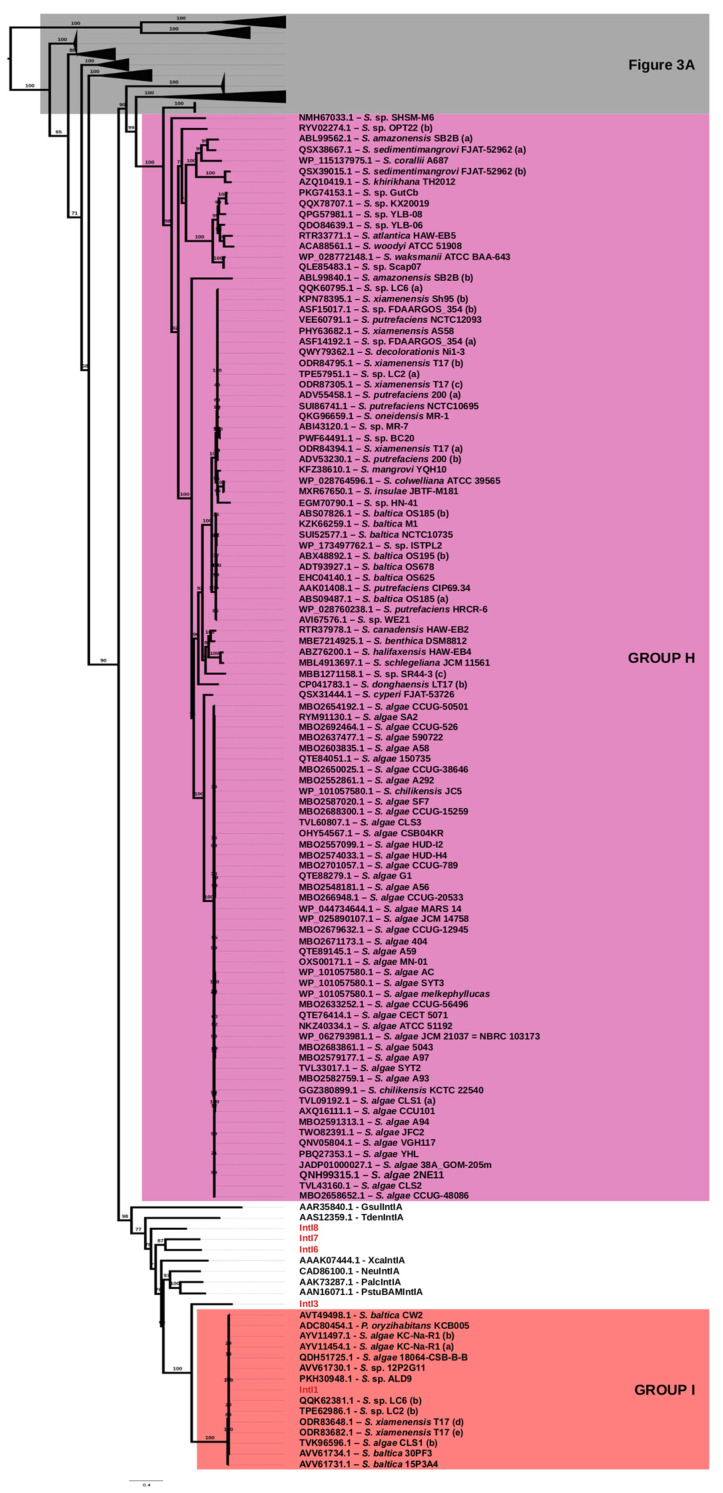

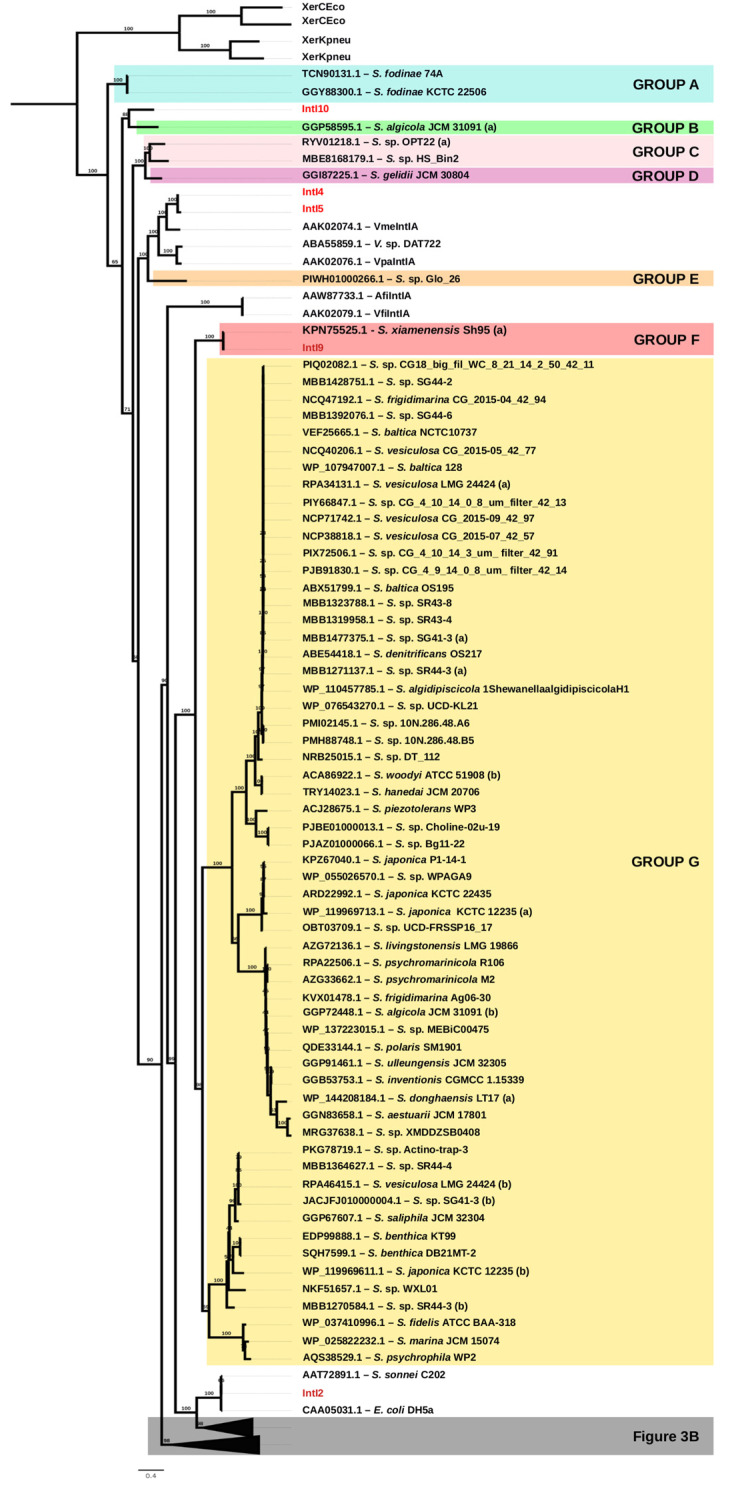
Phylogenetic tree of integron integrases found in *Shewanella* spp. (**A**) Depiction of branches from Groups A, B, C, D, E, F and G. (**B**) Depiction of branches from Groups H and I. Tree construction was done using the maximum-likelihood method with model LG + I + G4 and a bootstrap of 1000 replicates. IntI1 to IntI10 integron integrases are depicted in red. A detailed description of each group can be found in Table S3.

Phylogenetic trees were constructed with IQ-TREE v1.5.5 software [41] using the maximum-likelihood method with model LG+I+G4 and the ultrafast bootstrap parameters (1000 replicates) to evaluate the node support [42] (Figure 3). The integrase protein sequences included in the analysis were listed in Table S3, and corresponds to 182 integron integrases from *Shewanella* spp., 24 known integron integrases (IntI1: ADW78905.1, ADC80454.1; IntI2: ADH82153.1, CAA05031.1, AAT72891.1; IntI3: AAO32355.1; IntI4: AAD53319.1; IntI5: AAD55407.2; IntI6: AAK00307.1; IntI7: AAK00305.7; IntI8: AAK00304.1; IntI9: AAK95987.1; IntI10: CAC35342.1; XcaIntIA: AAAK07444.1; *V*. sp. DAT722, IntIA: ABA55859.1; VmeIntIA: AAK02074.1; VpaIntIA: AAK02076.1; AfiIntIA: AAW87733.1; VfiIntIA: AAK02079.1; GsulIntIA: AAR35840.1; TdenIntIA: AAS12359.1; NeuIntIA: CAD86100.1; PalcIntIA: AAK73287.1; PstuBAMIntI: AAN16071.1) and 4 XerC and XerD proteins sequences, which were used as outgroups. Sequence alignment was done using MUSCLE in MEGA X version 10.0.4 software (Molecular Evolutionary Genetics Analysis across computing platforms) [43].

### 2.5. Genetic Context of Integron Analyses

Genetic surroundings of integron integrase genes (n = 121) were analyzed in complete genomes (47/121) and in contigs with more than 1000 bp adjacent to an In0 or to a complete integron (74/121) using Geneious Prime v.2021.0.3 software (https://www.geneious.com), to visualize the integron integrases genomic context. SoptIntIA-like (*S. sp*. HS_Bin2) was also included as it was the only representative of Group C (Figure S1). Adjacent protein sequences were analyzed by blastp and tblastn using Genbank and Pfam databases. Insertion sequences (IS) were detected using the webserver from ISFinder (Figure S1). Genomes that did not have an available annotation (n = 18) were excluded except for *Shewanella* sp. Glo_26, since it was the only representative of Group E (Figure S1).

## 3. Results

### 3.1. Identification of Integrase Genes Encoded in Shewanella *spp.*

In order to evaluate the occurrence of integron integrases in *Shewanella* spp., we searched for the presence of the integrase gene (*intI*) in genomes available in GenBank until 1 April 2021 (81 complete and 223 draft genomes). We found 182 integrase genes in 158 genomes showing an occurrence of 52%. Fourteen sequences corresponded to incomplete genes, thus they were excluded from the analysis (Table S3). Alignment of the 182 IntI sequences resulted in the identification of all main residues (E121, K171, K219, W229, F233, G302, ALER215, and RHRY). K171, K219, W229, F233, H277, R280 were conserved in all integrases. E121 showed a high conservation in our dataset (96.1%; 175/182); however, some sequences had an E121Q substitution (3.8%; 7/182), which has been previously reported for marine integrons [44], and residue G302 was conserved in 99.4% of integrases (181/182) (Table S3). Regarding the motif ALER, we found that 95 out of 182 IntI (52.2%) had the canonical sequence, whereas 21 integrases had different motives, where SLIR was the most frequent variant (14.3%; 26/182) (Table S3 and Figure S2). L216 from the ALER motif was the single conserved residue in all but one sequence encoded in the animal gut isolate *S*. *waksmanii* (ATCC BAA-643). Last, the residue Y312 was conserved in all integrases, except for the one found in *S. corallii* A687 (Y312C). Overall, integron integrase sequences detected in this genus showed considerable differences that may reflect variations in the recombination processes.

We then evaluated the relationship among integrase proteins by phylogenetic analysis and primary sequence identity. The phylogenetic tree revealed a wide distribution of these enzymes along different *Shewanella* spp. showing an heterogeneous distribution (Figure 3A,B). Taking into account the cut-off values for the respective % of identity (% ID) at the protein level, we defined the same integrase type to those that have >95% ID; an IntI-like when values ranged from <95%–>70% ID; or a different IntI type when values were <70% ID (Figure 2 and Table S2). Based on this criterion, the data were classified in nine groups (from A to I). Identity values ranged from 29.6% to 70% among groups. Groups A to F comprised a few integrases (8/182), showing a limited occurrence of these variants in this genus. Since we found an unexpected diversity of integrases among *Shewanella* spp., we assigned each cluster using previous nomenclature [13,20,37,38]. Group A contained two integrases found in two strains of *S. fodinae* (SfoIntIA; TCN90131.1, GGY88300.1). The range of identity values within each Group can be observed in Figure 2 and Table S2. Group B consisted of an integrase in *S. algicola* JCM31091 (SalIntIA; GGP58595.1). Group C contained an integrase identified as SoptIntIA (*S. sp*. OPT22; RYV01218.1) and one SoptIntIA-like integrase (*S. sp*. HS_Bin2; MBE8168179.1). Group D and E consisted of a single integrase from *S. gelidii* JCM30804 (SgeIntIA; GGI87225.1) and *S. sp*. Glo_26 (SgIoIntIA; PIWH01000266.1), respectively. Group F contained an IntI9-like integrase previously reported in *S. xiamenensis* Sh95, located in an ICE from the SXT/R391 family (KPN75525.1) [7]. As seen in Figure 3, most integrases clustered into Groups G and H. Group G contained a large number of integrases divided in seven lineages (32.4%; 59/182) (Figure 3A). We identified these lineages as (i) SveIntIA (23 members) and SveIntIA-like (3 members); (ii) SpieIntIA (ACJ28675.1); (iii) SchoIntIA (two members; PJBE01000013.1, PJAZ01000066.1); (iv) SjaIntIA (five members); (v) SfriIntIA (seven members) and SfriIntIA-like (five members); (vi) SactIntIA (four members) and SactIntIA-like (six members); and (vii) SmarIntIA (WP_025822232.1) and SmarIntIA-like (two members; AQS38529.1, WP_037410996.1). Group H was the most abundant, with 102 members divided in seven lineages (56%; 102/182) (Figure 3B). Within this group we found the already described integrases SonIntIA (QKG96659.1) [34] and SamIntIA (ABL99562.1) [38]; however, our analysis showed a wider diversity. These lineages were identified as: (i) SshIntIA(NMH67033.1); (ii) SamIntIA (ABL99562.1) and SamIntIA-like (two members; QSX38667.1, WP_115137975.1); (iii) SfjIntIA (QSX39015.1); (iv) SkhiIntIA (AZQ10419.1); (v) SoptIntIB (RYV02274.1); (vi) SguIntIA (2 members) and SguIntIA-like (six members); and (vii) SonIntIA (17 members) and SonIntIA-like (70 members).

Last, Group I contained all IntI1 integrases (7.2%; 13/182) with a strong sequence conservation (identity > 99.1%) (Table S2 and Figure 3B). A few integrases were excluded from the analysis, i.e., integrase genes found in *S. xiamenensis* T17 (NGZL01000175.1_1) and *S. xiamenensis* CC4-7 (ALD16294.1), which were interrupted at the C-term end by IS26-bounded pseudo compound transposons identified as PTn-tet [45] and those found in *S. sp*. Shew256 (NAJR01000050.1) and *S. putrefaciens* SA70 (ODR83671.1) that were fragmented at the end of the respective contigs (Table S3).

Our analysis revealed the presence of 22 integrase gene types in *Shewanella* spp., 17 of which have not been described before.

### 3.2. Unique Distribution of Integrase Genes among Shewanella *spp.* Genomes

A detailed analysis of the distribution of the integrase genes from Groups A-E, G or H revealed that they were not ubiquitous among *Shewanella* spp. (Figure 3). Some integrase genes were found in different species of *Shewanella,* such as Sve*intIA,* which was found in *S. vesiculosa*, *S. frigidimarina* and *S. baltica* strains (Figure 3A, Group G). Most of these strain-specific integrase genes were found at a chromosomal location as typical CIs, except for Son*intIA* and Sve*intIA* which were embedded in plasmids (see below) (Table S3). As expected, MI integrase genes *intI1* and *intI9*-like were detected in different *Shewanella* spp., i.e., *intI9*-like in *S. xiamenensis*, and *intI1* in *S. algae*, *S. xiamenensis* and *S. baltica* strains (Figure 3B, Group I).

We also found that 45/85 (52.9%) genomes of *S. algae* from our dataset encoded a Son*intIA*-like integrase gene, while the remaining did not contain a homologous gene (Table S1). MLST analysis of all 85 *S. algae* genomes showed that neither the strains lacking Son*IntIA*-like nor those that have an integrase gene belonged to the same lineage (Table S1). *S. algae* strains were either distributed into 54 STs, 49 were singletons and five STs (1, 3, 33, 39 and 56) were shared between two or three strains; the remaining 25 strains did not have an assigned ST. Analysis of gene alleles did not show further clonal relationships among them. On the other hand, MLST analysis of *S. baltica* strains (n = 16) revealed that a few of them shared similar allelic profiles and carried either the same or different integrase gene types, supporting their potential LGT (Table S1).

Although several *Shewanella* spp. isolates encoded a single integrase gene, we found that some strains had two or more homologues (20/158; 12.6%) (Figure 3 and Table S3). Frequent combinations corresponded to (i) *intI1* with Son*intIA* or Son*intIA*-like (4/20), (ii) integrase genes exclusively from Group G (3/20), or (iii) integrase genes exclusively from Group H (5/20). Increased frequencies observed for these three combinations may be due to a higher incidence of integrase genes in our dataset.

We wondered whether the LGT of integrase genes may have also occurred from *Shewanella* strains to other genera. Thus, we looked for all integrase gene types (n = 22) from all nine Groups in other bacterial species. We found homologue genes for 14 of them with high identity sequence (% ID > 95) in isolates from *Alteromonadales*, *Vibrionales*, *Chromatiales*, *Oceanospirillales* and *Enterobacterales* orders (Table S4). For example, Sve*intIA*, Scho*intIA*, and Son*intIA* homologue genes belonging to different groups (G and H) were observed in a wide variety of strains of *Vibrio* spp. species. Noteworthy, Sglo*intIA* homologue genes were found in 115 *Vibrio* spp. strains from at least 12 different species. Similarly, Sact*intIA* homologue genes were identified in nine strains from different species of *Pseudoalteromonas* spp. (Table S4). Excluding MI integrase genes, only one homologue with high sequence identity (% ID > 93) was found in a plasmid, which corresponded to Sal*intIA* located in *Pseudoalteromonas* sp. Bsw20308 megaplasmid pPBSW1 (CP013139.1).

These findings evidenced complex evolutionary pathways of the novel integrase genes identified in *Shewanella* spp.

### 3.3. Integrase Genes from Shewanella *spp.* Are Not Niche Dependent

We investigated whether the distribution of integrase genes was related to the habitat from which each *Shewanella* spp. isolate was recovered. Therefore, we evaluated their association between the source of each isolate and the presence of an integrase gene. We first noticed that most integrase genes were found in strains recovered from sediments or aquatic niches (74/158; 46.8%), whereas it was less frequent to find integrase genes in bacteria isolated from human-associated niches (clinical samples, hospital environments or other impacted niches; 44/158; 27.8%) (Table S5). Bacteria from aquatic or sediment habitats together with those from animal samples showed the widest variety of integrase gene types (Figure S3, blue and green bars, respectively). Furthermore, two integrase gene types showed a broader dissemination in several habitats, corresponding to Son*intIA* from Group H and *intI1* from Group I.

A more limited diversity of integrase genes was noticed in bacteria recovered from clinical samples, which contained *intI9*-like, Son*intIA* (or Son*intIA*-like) and/or *intI1*. It is worth to mention that *intI1*-bearing bacteria were mostly isolated from human-associated samples (6/13); however, we also found that 4/13 were recovered from aquatic animals, which provides additional evidence of the role of animal reservoir and/or source in multidrug resistant (MDR) bacteria evolution. The impact of these reservoirs was also seen for Son*intIA* and Son*intIA*-like integrase genes, where 12 out of 81 were found in animal-associated bacteria.

### 3.4. Analysis of Integron/Cassette Systems in Shewanella *spp.* Genomes

In order to identify all gene cassettes in each integron, we first analyzed all genes located upstream of integrase genes found in complete genomes and contigs with >1000 bp surrounding those genes in *Shewanella* spp. (n = 97) and assessed whether they had the VR. Several genomes harbored more than one integrase gene resulting in 121 integrons. *attC* curation allowed us to define the VR of each integron and to identify all gene cassettes. Each potential gene cassette was analyzed by looking for key *attC* features, such as size (> than 59 bp), presence of core site (CS) and inverse core site (ICS) sequences (GTTRRRY and RYYYAAC, respectively). In addition, bottom strand DNA of each *attC* site was folded using the mfold software in order to confirm the presence of a canonical secondary structure [20]. This allowed us to detect other structures that could correspond to *attC* or *attC*-like site structures [12].

All Groups had integrons harboring gene cassettes within the VR, which suggests that all of them contained active integrases capable of gaining or losing gene cassettes and may contribute to *Shewanella* evolution. Furthermore, analysis of each integron showed that 58 of them had a VR with different gene cassettes, whereas 63 had an integrase gene without gene cassettes inserted at the *attI* site and therefore they were classified as In0 (Figure S1).

Several gene cassettes had two (n = 19) or three (n = 4) *orfs* in tandem associated to a single *attC* in their VR (i.e., *S. fodinae* strains 74A and KCTC 22506 in Group A, *S. gelidii* JCM 30804 in Group D, *S. japonica* UCD-FRSSP16_17 and *S. sp*. Actino-trap-3 in Group G, *S. amazonensis* SB2B, *S. khirikhana* TH2012, *S. xiamenensis* T17, *S. sp*. strains YLB-06, Scap07, LC6, LC2, WE21 and *S. algae* strains 150735, A59, CECT 5071, ATCC 51192, G1, A292, CCUG-789 in Group H; Figure S1; blue triangle). In addition, one integron contained a gene cassette lacking an *orf* suggesting that they may express a non-coding RNA (i.e., *S. inventionis* CGMCC 1.15339 in Group G; Figure S1, green triangle). The presence of two *orfs* in tandem in a single gene cassette or no *orf* in it has been previously reported for other integrons [20,46]; however, they had not been reported in previously characterized integrons from *Shewanella* spp.

Array analysis of the VRs revealed that *Shewanella* integrons may contain up to nine gene cassettes (Figure S2). Sequence analysis showed that their functions were quite diverse, encoding antimicrobial resistance mechanisms, as well as proteins involved in stress resistance, DNA repair, and cellular regulation, whereas most gene cassettes coded for proteins of unknown function (n = 141). Regarding the AMR gene cassettes, all of them were located in the VR of class 1 integrons, except for *aadA24*. These gene cassettes conferred resistance to different antibiotic families, i.e., aminoglycosides (*aadA1, aadA1e, aadA2*, *aadA6*, *aadA16, aadA24, aadB*, *aacC1*, *aac(6’)-Ib7Δ*), trimethoprim (*dfrA12, dfrA15*, *dfrA27*), rifampicin (*arr-2*, *arr-3*), chloramphenicol (*cmlA5*), beta-lactams (*bla*_VEB-1_, *bla*_OXA-10_), or quaternary ammonium salts (*qacE* and *qacH*).

We also found group II introns (GII), which are ribozymes capable of retrotransposing to new regions in a genome, inserted at the *attC* site of gene cassettes *dfrA15*, *qacE, aadA24* and *aadA1e*. Invasion of AMR gene cassettes by these retroelements were previously reported for class C-attC GII introns [47,48] (Figure S2). Class C-attC GII introns were found in gene cassettes inserted in integrons from groups A, F, G, H, and I, suggesting that independent recombination events may have occurred. In addition, two integrons contained other GII introns corresponding to chloroplast Cl1 and bacterial E classes (i.e., strains *S. putrefaciens* 200 and *S. saliphila* JCM 32304, Figure S2). While some GII introns invaded AMR gene cassettes (i.e., *dfrA15* from strain Sh95), most ribozymes were inserted within gene cassettes encoding proteins of unknown function (i.e., in strain *S. fodinae* 74A from group A, *S. baltica* NCTC10737, *S. livingstonensis* LMG 19866, *S. saliphila* JCM 32304 from Group G and *S. sp*. strains LC6, LC2, FDAARGOS_354, *S. putrefaciens* NCTC12093, *S. xiamenensis* T17 (a,c) from Group H; Figure S2). Class C-attC GII introns are known for inserting into the target DNA in the opposite orientation to gene cassettes; however, we noticed that the ribozyme located in the genome of *S. algae* KC-Na-R1 was in the same orientation, which suggests that retrotransposition might have followed a different invasion process (Figure S2, *S. algae* KC-Na- R1 (b), Group I).

The VRs of several integrons were also interrupted by a wide variety of ISs, which belonged to families IS*91* (IS*91*-like, IS*Shvi3*) (n = 3), IS*1595* (IS*1595*-like, IS*Sod11*) (n = 2), and IS*110* (IS*Sde13*, IS*110*-like) (n = 5) (Figure S2). Most ISs were inserted within coding regions; however, it has been reported that IS*1111* elements are capable of invading the *attC* site of gene cassettes similarly to class C-attC GII introns [49]. Our analysis did not detect ISs interrupting *attC* sites, instead, we found an IS*91*-like element, named IS*Shvi3*, in the VR of the integron from *S. sp*. Choline-02u, which was adjacent to an *attC* site yielding a new gene cassette structure.

**Figure 4:**
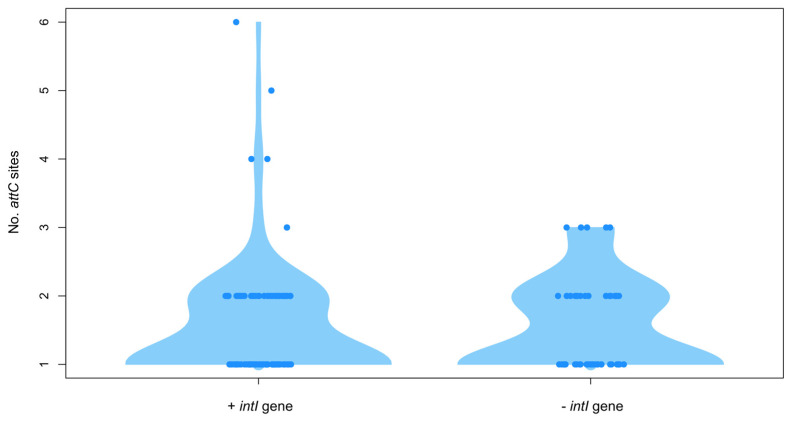
*attC* sites found in complete genomes of *Shewanella* spp. *attC* sites in CALINs and SALINs found in *Shewanella* spp. complete genomes with (left) and without (right) the *intI* integrase genes.

Previous studies have shown that gene cassettes were not only located within the VRs, but also, they can form clusters of *attC* sites lacking integron–integrases (also known as CALINs) [12]. In this regard, we observed a peculiar amount of *attC* sites that were found in distal regions of integrase genes. These CALINs were present in most *Shewanella* spp. strains. A detailed analysis showed that complete genomes that carried *intI* genes and CALINs (n = 33) were more frequent than genomes with CALINs and without *intI* genes (n = 26). Genomes containing CALINs (n = 59) had two (n = 47), three (n = 7), four (n = 2), five (n = 1) and up to six (n = 1) *attC* sites, each adjacent to an *orf* (Figure 4 and Table S6). Furthermore, we found single orphan gene cassettes (n = 115) classified as SALINs (single *attC* sites lacking integron-integrases; Alonso et al., in revision), scattered around several genomes (n = 41). In addition, in the presence of integrase genes CALIN arrays showed a slight increment on the number of *attC* sites per array, (an average of 1.51 *attC* sites/array with *intI* vs. 1.33 for genomes w/o *intI*).

This analysis exposes the wide diversity of gene cassette arrays and MGEs that are part of integron systems within a single genus.

### 3.5. Genetic Context of Shewanella *spp.* Integrons

We then studied the genetic context of 121 integrons found in *Shewanella* spp. We looked up all genes located downstream of the integrase gene in all complete genomes and in contigs with enough information that allowed us to assess their genetic background (length > 1000 bp). We analyzed 121 integron sequences, found in 47 complete and 74 draft genomes (Figure S2). At a first glance, we noticed genes encoding proteins with a wide variety of functions. Genetic contexts were very diverse even within the same *intI* group, i.e., Sve*intIA* genes were adjacent to hypothetical proteins PMH88747.1, PMI02144.1, VEF25666.1 and ABX51800.1, as well as to the peptidase M28 (MBB1477376.1) and NADH dehydrogenase (ABE54417.1). In a few instances, we found some conservation where homologous *intIA* genes from the same group were adjacent to the same gene (Sfo*intIA* from Group A; Sfri*intIA* and Sact*intIA* from Group G and Son*intIA*-like from *S. algae* and *chilikensis* from Group H). Interestingly, we also found integrase genes from different groups located downstream of the same gene. For example, Spie*intIA* from *S. piezotolerans* WP3, Scho*intIA* from *S. sp*. Choline-02u-19 (Group G) and Sgu*intIA* from *S. sp*. KX20019 (Group H) were located downstream of a gene coding for the subunit α of the tryptophan synthase α2β2 (TrpA; WP_202285266) (Figure S2).

ISs and other MGE-related genes were found downstream and/or upstream of integrons (Figure S2, depicted with orange arrows). Incidence of these elements was higher in Group I, which encompass all class 1 integrons embedded in transposons that most likely are harbored in different plasmids (Figure S2).

**Figure 5:**
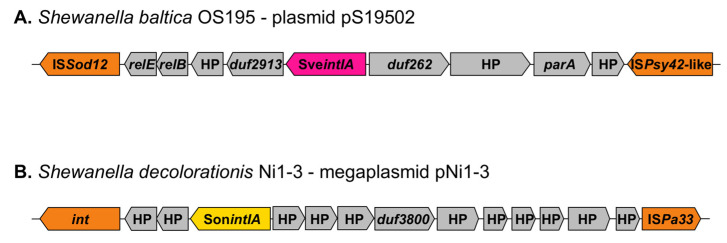
Genetic context of integrase genes found in plasmids from *Shewanella* spp. (**A**) Integrase gene Sve*intIA* encoded in plasmid pS19502 from *S. baltica* OS195 (CP000893.1). (**B**) Integrase gene Son*intIA* encoded in megaplasmid pNi1-3 from *S. decolorationis* Ni1-3 (CP076856.1).

All integrase genes had a chromosomal location, except for Son*intIA* and Sve*intIA,* that were found in plasmids; both integrons lacked gene cassettes and were classified as In0. Son*intIA* (QWY79362.1; Group H) was encoded in the megaplasmid pNi1-3 (CP076856.1) from *S. decolorationis* Ni1-3, and Sve*intIA* (ABX51799.1; Group G) was encoded in plasmid pS19502 from *S. baltica* OS195 (CP000893.1). While Son*intIA* was in the vicinity of an IS*Pa33*, Sve*intIA* was surrounded by IS*Sod12* downstream of the integrase gene and IS*Psy42*-like upstream of it (Figure 5). Comparative analysis of both plasmids harboring these integrons showed that they do not share homology nor have similar IS elements.

Although we observed a limited correlation among integrase genes and their respective flanking sequences, the wide variety of MGEs identified in the present study suggests that they may have been involved in several processes of LGT leading to the emergence of novel MIs.

## 4. Discussion

The study of integrons in the genus *Shewanella* revealed unique features that contribute to increasing our knowledge regarding their dissemination and their relevance in nature. Our in silico approach provides a detailed analysis of the genetic surroundings of integrase genes that widen our understanding on integron evolution from CIs towards MIs within the genus, as well as their consequent role in the adaptation of *Shewanella* spp. to new niches leading to their evolution to MDR in hospital settings.

To properly detect all candidate integrase genes, we analyzed the extensive work of previous groups and established an effective criterion based on the integron integrase phylogenetics and sequence analysis [19,20]. Our study led us to the identification of a surprising amount of integrase gene types within a single genus. In addition to the already reported integrase genes in *Shewanella* spp. [34,38], we found 17 new types clustered in different groups (Table S3). Novel integrase genes from *Shewanella* spp. showed key differences at the protein sequence level, such as the highly variable ALER motif, with up to 21 differences, which might directly impact on the enzyme activity [18,19,20].

The richness of integrase gene types in the environment has been previously reported; however, most studies used culture-independent techniques, where integrons cannot be assigned to a specific bacterial species or genus [36,50,51,52,53,54]. In *Shewanella* spp., integrase gene types were found scattered and some of them were even shared by different species (Table S4). The in silico analysis at the strain level allowed us to identify some genomes that harbored more than one integron, including both chromosomal and/or mobile integrase gene types. For instance, *Shewanella* sp. SR44-3 encoded integrase genes Sve*intIA*, Sact*intIA*-like, Son*intIA*-like, belonging to Groups G and H, or *S. xiamenensis* T17, which had five integrase genes corresponding to two *intI1* and three Son*intIA*. To the best of our knowledge, the co-existence of different chromosomal integrase genes in the same strain has not been previously found.

In addition to evidencing the ability of *Shewanella* spp. strains to acquire MI integrase genes, such as *intI1* and *intI9*, dynamic LGT events of the novel chromosomal integrase genes can be inferred. Integrase genes with > 95% identity from Groups G and H were found scattered in several strains of *Alteromonadales*, *Vibrionales*, *Chromatiales*, *Oceanospirillales* and *Enterobacterales* (Table S4). Therefore, it remains a crucial question if these novel integrase genes can be assigned to a particular species or if they are subjected to continuous processes of LGT.

Regarding the VR of integrons, we found that they contained on average one and up to nine gene cassettes (Figure S1). This feature resembles the VR size of MIs and CIs from *Xanthomonas*, *Nitrosomonas* or *Geobacter*, among others, but they differ from the VR of *Vibrio* spp., which usually contain around 100 gene cassettes [13,30,37,55]. We also found that the 52% (63/121) of integrons did not harbor any gene cassette in the VR, evidencing another feature of CIs in the genus *Shewanella.* Analysis of the array content of gene cassettes did not show any particular association in regards to *Shewanella* species, integrase gene type or habitat, which suggests that these integrons followed independent evolutionary pathways. While CI gene cassette arrays from *V. cholerae* may be conserved within the species [56], this is not the behavior observed for those from *Shewanella* spp.

Although there are different criteria to classify integrons [20,21,24,25,26], a hallmark of CIs is that they co-evolve with their host over long periods allowing to identify its ubiquity within a given species or lineage [21,25,56]. In this regard, *intIA* from *V. cholerae* represents the paradigm of chromosomal sedentary integrase genes [21,30]. On the other hand, MIs have a continuous mobilization by LGT events to several hosts which is evidenced by the great variety of species where they can be found as class 1 integrons in clinical and environmental strains [12,13]. In this regard, integrase genes in the genus *Shewanella* showed distribution patterns not yet described in other genera.

Our analysis revealed that the integrase genes in *Shewanella* spp. followed three different evolutionary pathways. The first one corresponds to the typical CI co-evolving with the host, with the chromosomal integrase gene located at the same loci. For instance, Son*intIA*-like was found in different *S. algae* clones at the same chromosomal location- suggesting the possible co-evolution between integrase genes and some strains similarly to CIs from *V. cholerae*. Our phylogenetic studies reinforce the hypothesis that Son*intIA*-like from *S. algae* has likely evolved from a common ancestor. Although not all *S. algae* genomes encoded this gene, it is also probable that some strains have lost their integrase genes after independent evolutionary events. CIs with signs of sedentarism within the chromosome may be present in other species of *Shewanella*; however, since there are very few genomes sequenced from several species, we do not have enough data to confirm this hypothesis.

The second evolutionary pathway corresponds to CI integrase genes spread among different strains of *Shewanella* spp. Integrase genes were not ubiquitous within any species and they seem to be strain-specific, most of them located at different sites throughout the *Shewanella* chromosome, evidencing continuous processes of gain and loss along time (Table S3 and Figures S1 and S2). Accordingly, only six out of 10 complete genomes of *S. baltica* had a CI integrase gene at different loci (Table S3 and Figure S1). In addition, several CIs were adjacent to various ISs, which may have contributed to integron dissemination; however, their mechanism of insertion or deletion is unknown. Likewise, LGT of integrons has also been proposed for *Vibrio fischeri*, *Shewanella denitrificans*, *Nitrosococcus mobilis*, and *Xanthomonas* spp. [21].

The third evolutionary pathway corresponds to MIs evolution and dissemination. Our work led to the identification of molecular features of integrons that could reflect the acquisition of novel MIs within the genus. Two integrase genes commonly found in the host chromosome, Son*intIA* (from Group H) and Sve*intIA* (from Group G), were found in plasmids flanked by MGEs which may have contributed to their mobilization. Noteworthy, both integrase genes were also found in other distant genera, supporting the scenario of a possible transition from CIs to MIs. Spread of homologues of Son*intIA* with > 93% identity and 100% coverage were found in the chromosome of *Vibrio plantisponsor* LMG 24470 and in diverse strains of *Pseudoalteromonas piscicida*, *Pseudoalteromonas* sp., *Vibrio fluvialis* and *V. cholerae* (Table S4). Similarly, homologues to Sve*intIA* with 100% identity and coverage were found in the chromosome of *Vibrio metschnikovii* 07-2421, *Vibrio alginolyticus* VA181, *Vibrio* sp. E4404 and in diverse strains of *V. fluvialis*. In addition, the chromosomal integrase gene Sal*intIA* from *Shewanella algicola* JCM 31091 was found located in the megaplasmid pPBSW1 (CP013139.1) from *Pseudoalteromonas* sp. Bsw20308. Although we cannot define which strain acquired Sal*intIA*, our results reflect the transferability of these integrase genes. This unique pattern of mobilization of chromosomal integrase genes found from and to the genus *Shewanella* explores a scenario in which CIs may be commonly shared among different bacteria within a niche. These novel MI integrase genes may be reproducing the behavior of other MIs circulating among different genera in the clinical environment, while providing to each recipient strain the possibility of adapting to new niches thanks to the pool of gene cassettes available in bacterial genomes. Some strains of *Shewanella* spp. have shown the capability to acquire different MGEs harboring class 1 and 9 integrons [7,57], revealing their potential to evolve towards a MDR phenotype. Altogether our study shows that *Shewanella* spp. provides a scaffold where different types of integrons can co-exist, thrive and evolve.

On the other hand, integrase expression can be activated when bacteria encounter antimicrobial agents, giving rise to processes of excision and insertion of gene cassettes [58]. Recently, it has also been demonstrated that bacteria exposed to an increasing amount of antimicrobial agents in the presence of a working integrase were able to reorganize useful AMR gene cassettes from the last position to a top position [59]. In this scenario, the dissemination of Son*intIA* or Sve*intIA* located in plasmids to MDR isolates harboring AMR gene cassettes could seriously increase the antimicrobial resistance burden.

As a hallmark of the genus, we observed that integrons followed complex evolutionary pathways. Our study showed that *Shewanella* spp. can acquire and disseminate integrase genes that may lead to the emergence of novel MIs. The substantial diversity of gene cassettes found in the VR, and their frequent association with MGEs suggest a constant evolution and adaptation of the host, which probably responds to environmental niche changes and the composition of each microbial community. Furthermore, it must be taken in account that AMR gene cassettes found in hospital settings can be recruited from environmental CIs [13,26]. Since there is a clear link between the *intI1* gene and the dissemination of AMR in environmental and nosocomial niches, it is possible to assume that the considerable amount of integrase genes found in *Shewanella* spp. including novel MIs, poses a scenario in which a new integron system may emerge and contribute to the MDR threat. Active participants in these processes may include *S. algae* and *S. xiamenensis* species, which are opportunistic pathogens that can disseminate in several niches and cause serious infectious diseases.

## Data Availability

Not applicable.

## Supplementary Materials

The following are available online at https://www.mdpi.com/article/10.3390/microorganisms10061102/s1, Table S1. Complete Data Set. Table S2. Similarity and Identity of integron integrases found in *Shewanella* spp. Table S3. Additional information of integron integrases found in *Shewanella* spp. Table S4. *Shewanella* integrases found in other genera. Table S5. Source of *Shewanella* spp. isolates harboring integron integrases. Table S6. CALINs in complete genomes of *Shewanella* spp. Table S7. Description of genes and gene cassettes. Figure S1. Genetic context of *intI* genes in *Shewanella* spp. Figure S2. ALER motives identified in the different integron integrases found in *Shewanella* spp. Figure S3. Source of *Shewanella* spp. isolates harboring various integron integrase gene types.
