# Supplementary figures and images for: Novel Mobile Integrons and Strain-Specific Integrase Genes within Shewanella spp. Unveil Multiple Lateral Genetic Transfer Events within The Genus
Source: Microorganisms. 2022 May 26;10(6):1102. doi: 10.3390/microorganisms10061102 (PMC9229058; doi:10.3390/microorganisms10061102)

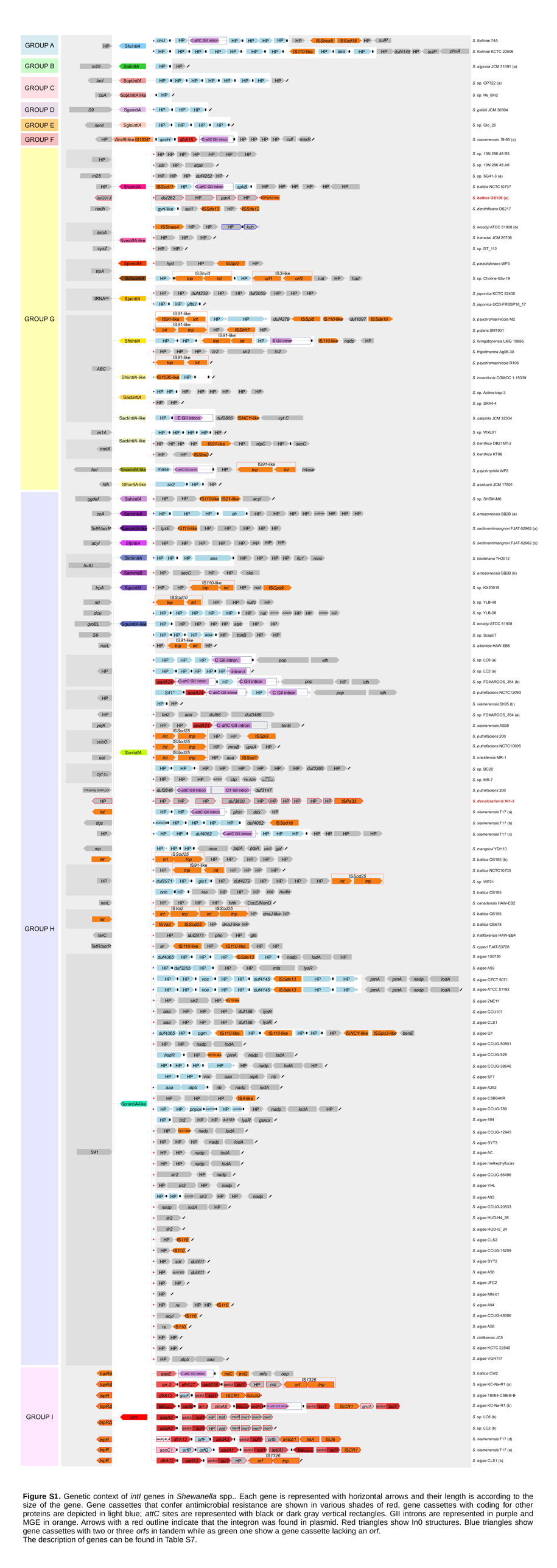

Supplement: Supplementary file 1 [file microorganisms-10-01102-s001.zip › Figure S1. Genetic context of intI genes in Shewanella spp..tif]

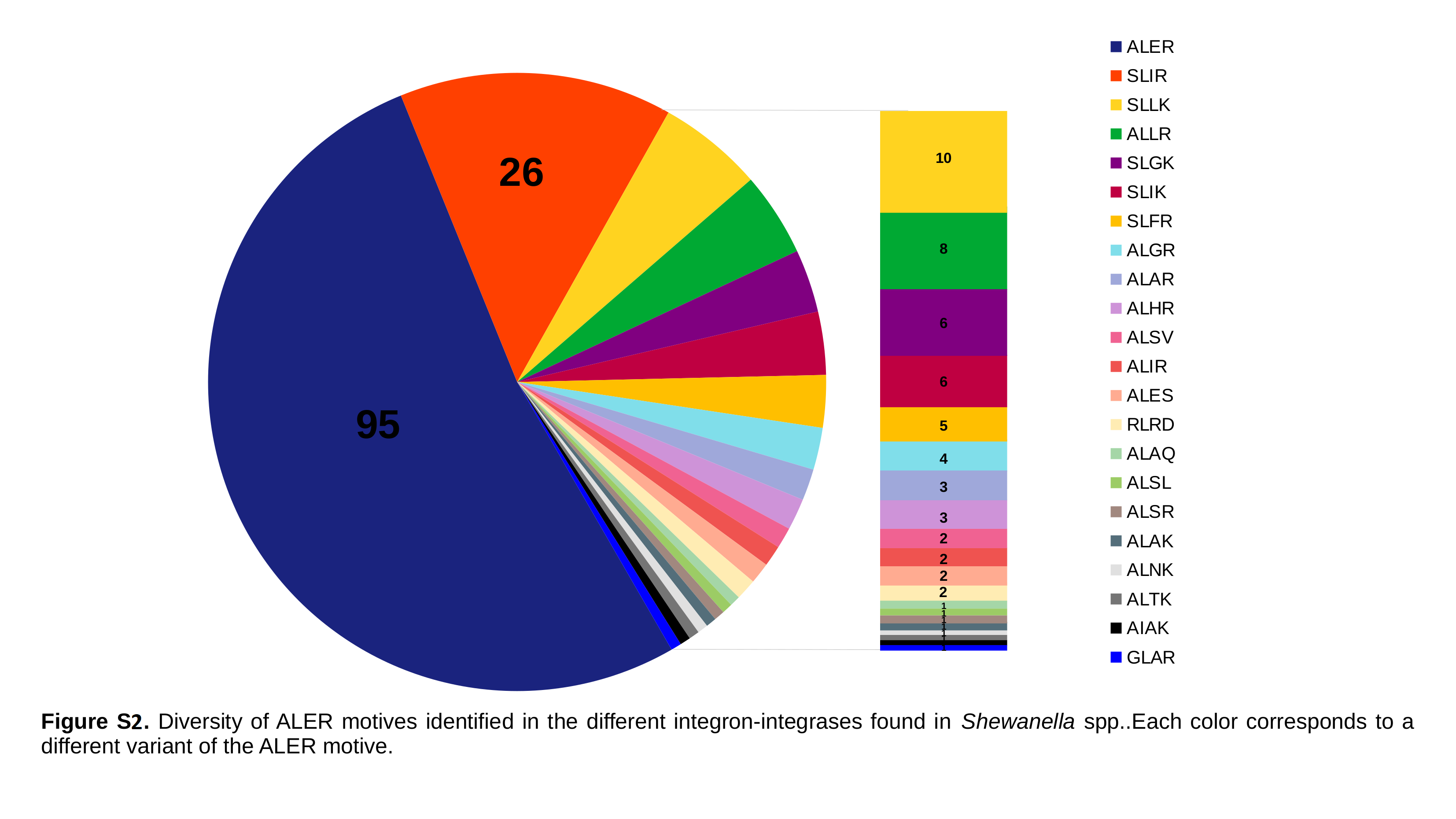

Supplement: Supplementary file 1 [file microorganisms-10-01102-s001.zip › Figure S2. ALER motives identified in the different integron integrases found in Shewanella spp..tif]

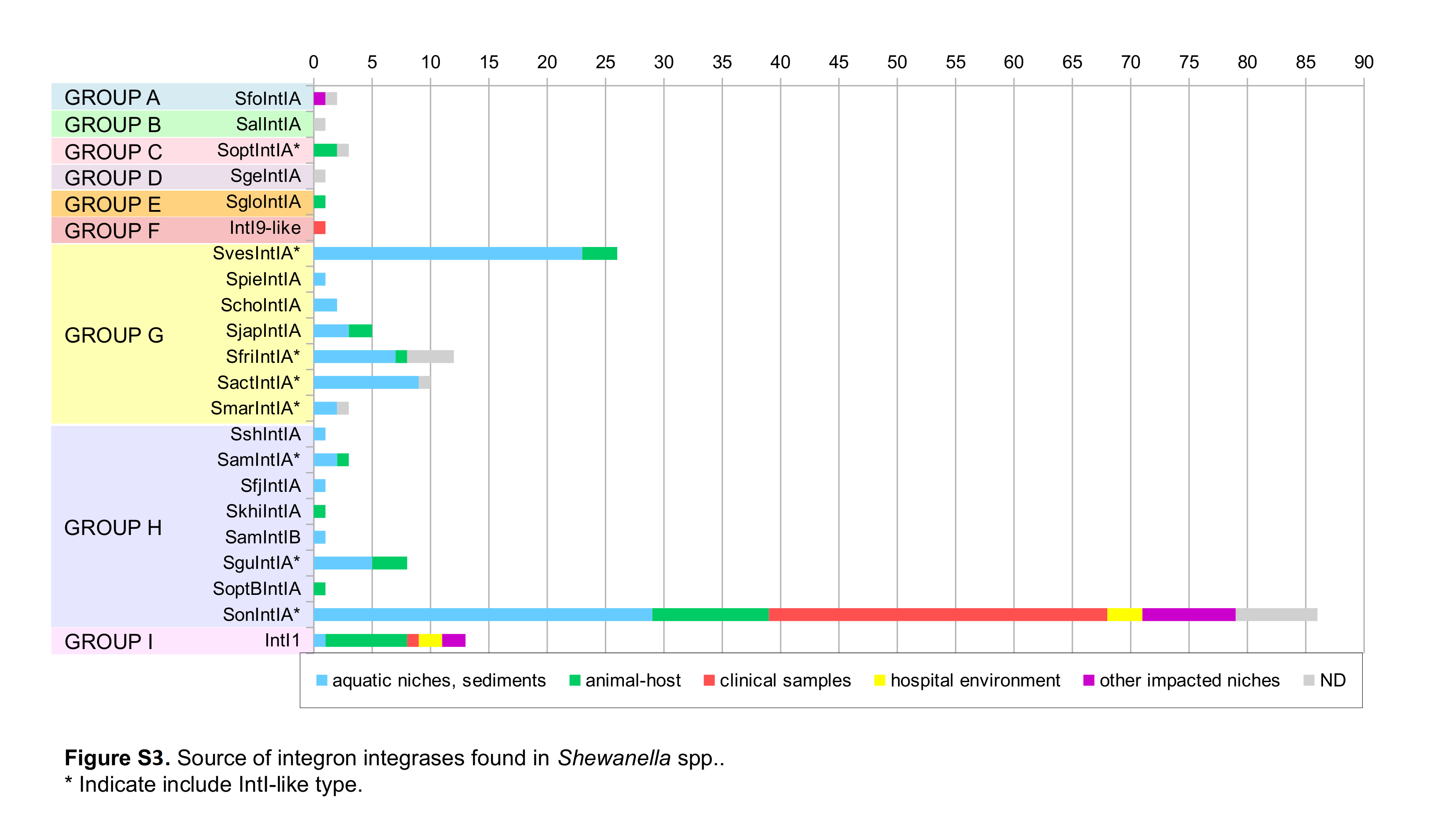

Supplement: Supplementary file 1 [file microorganisms-10-01102-s001.zip › Figure S3. Source of Shewanella spp. isolates harboring various integron integrase gene types..tif]
